# Editorial: Cognition and language contact: interdisciplinary and multidisciplinary approaches

**DOI:** 10.3389/fpsyg.2024.1425761

**Published:** 2024-05-27

**Authors:** Andrew K. F. Cheung

**Affiliations:** Department of Chinese and Bilingual Studies, Hong Kong Polytechnic University, Kowloon, Hong Kong SAR, China

**Keywords:** cognition, interpreting, translation, interdisciplinary, language

The purpose of the Research Topic titled “*Cognition and language contact: interdisciplinary and multidisciplinary approaches*” was to delve deeper into the complex interplay between human cognition and language. Over recent decades, the enigmatic ways in which the human mind processes multiple languages have captivated researchers (Kroll and Groot, 2005; Schwieter and Tokowicz, 2015). By drawing on diverse fields such as linguistics, cognitive psychology, neuroscience, and computer science, researchers have gained more and more understanding of the intricate mechanisms that underpin language processing (Kroll and Groot, 2005; De Zubicaray and Schiller, 2019).

The interdisciplinary, or even multidisciplinary approach, is undertaken in the Research Topic. Out of the eight articles featured, six specifically explore language processing within the context of translation and interpreting (T&I), which are considered as outcomes of various causes within the translators/interpreters' minds and outside it (Chesterman, 2017). The T&I process demands sophisticated cognitive skills, such as simultaneously grasping source and target texts to accurately transfer meanings and styles (Jin and Cheung, 2023), remembering and retaining information, analyzing the structure and meaning of the source language while avoiding linguistic interference (Ma and Cheung, 2020), making rapid decisions under time constraints (Liu et al., 2023), maintaining focus during lengthy tasks, efficiently managing attention, tackling problems (Cheung, 2019), devising solutions, and using metacognition to regulate cognitive processes (Gile, 2009; Grosjean, 2010; Schwieter and Ferreira, 2017; Dong and Li, 2020). The complexities of language processing in TandI offer a unique lens through which to examine cognition and languages (Song and Cheung, 2019; Wu et al., 2021). Among the six articles, Han et al., Chang and Chen, and Song et al. focus on the I/T process. Han et al. differentiated between simultaneous and consecutive interpreting by analyzing interpreters' “translanguaging moments.” The analysis reveals a generally positive attitude toward the two interpreting tasks.

Chang and Chen focused on “translation asymmetry,” using pupillometric data and a machine-learning algorithm to classify the bilateral translation. The classification has achieved high accuracy scores, indicating that the pupillometric data can effectively predict the direction in translation. Song et al. studied direction of interpreting with a focus on interpreters' executive competencies assessed through psychological tests among a group of unbalanced bilinguals. Results suggest the types of executive functions interact with the direction of interpreting to have an impact on the interpreting performances.

Other three studies conducted by Huang et al., Liu and Dou, and Ren and Wang, employ a corpus-assisted and product-oriented approach. All three focus on simultaneous interpreting—a highly demanding cognitive task. Huang et al. investigate how phrasal frames are used as a strategy to cope with different amounts of cognitive loads varied by complexities in task processing (interpreted and non-interpreted tasks) and the directionality in interpreting. They conclude that interpreters in retour interpreting (into-L2 interpreting) encounter the most cognitive loads and use the largest number of phrasal frames. Liu and Dou and also address cognitive loads and found that simplification typically occurs only to alleviate task-related pressures. Retour interpreting does not necessarily result in higher cognitive load or simplification. Ren and Wang examine the use of metadiscourse markers in both parallel corpora with Chinese as the source language and English as the target language, and a comparable corpus with interpreted and original English. Results indicate simultaneous interpreters use metadiscourse markers to implement various strategies. Wu et al., on the other hand, adopt the corpus-assisted approach to study bilingual processing among second language learners and native speakers. They find proverb variations reflect learners' creative use of language and the ability to adapt language use to fit the context.

The last study, by Kim and Nam, investigates brain function in language processing. They employ a visual half-field paradigm to explore how the brain's hemispheres respond to different language processing tasks. Participants are presented with letter strings to either the left or right visual field to determine how each hemisphere processes language. They highlight the dominant role of the left hemisphere in high-demanding tasks.

Collectively, the studies in this Research Topic complement each other by exploring various linguistic phenomena and cognitive mechanisms. They illustrate the benefits that by combining perspectives from diverse fields, researchers can approach the study of language processing from multiple angles to uncover the interplay between linguistic knowledge, cognitive processes, neural mechanisms, and computational models. This interdisciplinary collaboration allows for a more comprehensive and nuanced understanding of how language is processed. We hope this Research Topic will inspire further interdisciplinary research on translation and interpreting.

## Author contributions

AC: Writing – original draft, Writing – review and editing.
